# Silicon fertilization in maize increases attractiveness of nocturnal herbivore‐induced plant volatiles to *Spodoptera frugiperda* natural enemies

**DOI:** 10.1002/ps.8768

**Published:** 2025-03-12

**Authors:** Patrícia Pereira, Tiago Morales‐Silva, Rosamara Souza Coelho, Marvin Pec, Kamila Emmanuella Xavier Azevedo, Arodí Prado, José Maurício Simões Bento, Rosangela Cristina Marucci, Bruno Henrique Sardinha de Souza, Maria Fernanda Gomes Villalba Peñaflor

**Affiliations:** ^1^ Departamento de Entomologia Universidade Federal de Lavras (UFLA), Campus Universitário Lavras Brazil; ^2^ Instituto Capixaba de Pesquisa Assistência Técnica e Extensão Rural (INCAPER, CPDI Norte) Linhares Brazil; ^3^ Departamento de Entomologia e Acarologia Escola Superior de Agricultura “Luiz de Queiroz” (Esalq/USP), Campus Universitário Piracicaba Brazil

**Keywords:** fall armyworm, plant defenses, olfactory behavior, predatory earwig, silicic acid, tritrophic interactions

## Abstract

**BACKGROUND:**

Silicon (Si) fertilization has been well‐documented to enhance plant resistance against insect pests by increasing the abrasiveness and toughness of leaf tissues. Additionally, Si also interacts with the jasmonic acid pathway, which modulates antiherbivore induced defenses, including the emission of herbivore‐induced plant volatiles (HIPVs) that attract natural enemies. In this study, we examined the influence of Si fertilization on the attractiveness of nocturnal HIPVs from maize plants infested with the fall armyworm (FAW), *Spodoptera frugiperda* (JE Smith), to the predatory earwig *Doru luteipes* (Scudder).

**RESULTS:**

In laboratory assays, we found that Si fertilization did not alter the attractiveness of nocturnal constitutive volatiles to the nocturnal predator *D*. *luteipes*. However, upon infestation with FAW larvae, Si‐fertilized plants emitted a volatile blend that was more attractive to *D. luteipes*. Although the composition of HIPVs emitted by non‐fertilized and Si‐fertilized plants was similar, the terpene neryl acetate was exclusively detected in the HIPV blend from Si‐fertilized plants. Tests with synthetic neryl acetate demonstrated that the terpene alone was attractive to the earwig at the specific concentration found in the HIPV blend emitted by Si‐fertilized plants.

**CONCLUSION:**

This study demonstrates that Si fertilization primes indirect defenses by specifically increasing the amount of neryl acetate in the HIPV blend, which is responsible for the enhanced attractiveness to the predatory earwig. Thus, Si can act as a priming agent of indirect plant defenses, potentially increasing the recruitment of the predatory earwig once plants are infested by FAW, thereby contributing to suppress insect pest populations. © 2025 The Author(s). *Pest Management Science* published by John Wiley & Sons Ltd on behalf of Society of Chemical Industry.

## Graphical abstract

Silicon (Si) fertilization enhances the attractiveness of plant volatiles emitted by fall armyworm‐infested maize plant to the predatory earwig *Doru luteipes* by triggering the emission of the terpene neryl acetate.

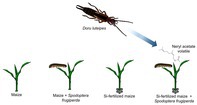

## 1 INTRODUCTION

Crop protection against insect pests often relies on chemical pesticides to minimize yield losses. However, these chemicals can pollute the environment and harm non‐target organisms, including natural enemies and pollinators, which provide essential ecosystem services in agroecosystems.^1^ In the current context of a global climate crisis, with drought and extreme temperatures limiting crop production and threatening food security, biostimulants have emerged as a promising tool to enhance plant health and confer protection against both abiotic and biotic stresses, providing a sustainable alternative for agriculture.^2^ Among them, silicon (Si) has drawn increasing attention due to its ability to enhance plant tolerance to a wide range of abiotic stresses, such as salinity, drought, high temperatures, ultraviolet (UV) radiation, toxic heavy metals and excessive minerals, as well as to biotic stressors, including pathogens and arthropod herbivores.^3^

Despite its benefits, supplementation with soluble Si is limited by the plants' ability to uptake it through specific transporters and accumulate the mineral in their tissues. Crop plants from the Poaceae family, such as rice, maize, wheat, oat and sugarcane, are among those with the highest Si accumulation capacity and benefit the most from it.^4^, ^5^ The formation of a mechanical barrier through the deposit of Si in plant epidermal cells has been regarded as the primary mechanism underlying the benefits.^6^ For instance, in terms of plant resistance to insects, the greater abrasiveness and hardness of the leaves in Si‐fertilized plants lead to mandible wear and reduced digestibility for pest insects.^7^, ^8^, ^9^, ^10^ However, the mechanical barrier is not the sole mechanism of protection, as Si accumulation also interacts with plant metabolism, influencing expression of stress‐responsive genes, enzymatic activity, and modulating plant signaling pathways that enhance the plant's ability to cope with stressors.^11^, ^12^, ^13^

Jasmonic acid (JA) plays a pivotal role in regulating the synthesis of herbivore‐induced plant defenses, with its activation initially triggered by mechanical damage.^14^ Fertilization with Si has been shown to increase levels of this phytohormone in rice plants, contributing to the enhanced herbivore‐induced plant response of Si‐fertilized plants to insect herbivory.^15^ The mechanism by which Si interacts synergistically with JA appears to involve a physical stimulus, due to its accumulation in plant tissue, that activates the JA signaling pathway^16^, ^17^ Consequently, Si fertilization can enhance plant defenses against herbivores, affecting them either directly – through the production of antinutritive, toxic, repellent, or unpalatable secondary metabolites – or indirectly, by inducing herbivore‐induced plant volatiles (HIPVs) that attract natural enemies of herbivores.^3^, ^18^ This occurs because Si accumulation can either prime or elicit JA‐modulated defenses, depending on the plant species.

While primed plants respond defensively more rapidly and vigorously to insect herbivore infestation,^19^ elicitation triggers the synthesis of defensive compounds even before insect infestation. For instance, Si accumulation in rice does not induce defenses but primes both direct and indirect antiherbivore defenses. The priming state of Si‐fertilized rice plants results in the production of elevated levels of secondary metabolites against caterpillars and the emission of a more attractive composition of volatiles to parasitoids at the onset of herbivory, compared to non‐fertilized plants.^13^, ^15^, ^20^ However, in wheat, Si deposition induces indirect defenses, making non‐infested plants attractive to natural enemies due to the constitutive emission of the volatile terpene geranyl acetone, which can negatively impact the ability of natural enemies to locate their hosts or prey.^21^ Unlike the priming effect in rice, Si‐induced volatile emissions from non‐infested wheat plants can mislead natural enemies, and the lack of reward associated with ecologically significant signals may alter their subsequent attraction to infested plants, potentially hampering the efficacy of biological control.^22^

Studies in a few monocot and dicot crops, such as sugarcane, cucumber, and beans, have reported increased recruitment of natural enemies in Si‐fertilized areas.^23^, ^24^, ^25^ However, the mechanisms underlying these effects have been scarcely studied and, as mentioned earlier, may result from a priming or elicitation effect on plant defenses induced by Si.^20^, ^26^, ^27^ In a previous study by our group,^26^ Si fertilization in maize negatively affected the biology and behavior of the fall armyworm (FAW) *Spodoptera frugiperda* (JE Smith) (Lepidoptera: Noctuidae), while increasing the attractiveness of HIPVs, but not constitutive volatiles, to the pirate flower bug *Orius insidiosus* (Say) (Hemiptera: Anthocoridae), a predator of eggs and neonate larvae. These findings suggest a priming effect of Si in maize but the chemical composition of the plant emissions was not characterized in this study.

Alternative control measures that can be incorporated into integrated pest management (IPM) programs for FAW in maize, one of the most widely produced agricultural commodities globally, are urgently needed. In the Americas, FAW in maize is primarily managed using chemical insecticides and transgenic maize; however, these control measures often quickly lead to the selection of resistant pest populations.^28^ Consistently, Si fertilization has shown positive results in reducing FAW injury in maize fields, with various modes of application and Si sources proving effective.^29^, ^30^, ^31^ Since Si fertilization can enhance the efficacy of biological control agents, it is important to investigate its effects on FAW's natural enemies. In South America, the predatory earwig *Doru luteipes* (Scudder) (Dermaptera: Forficulidae), a generalist nocturnal predator, is an important biological control agent of FAW eggs and larvae due to its voracity and prevalence in crops throughout the growing season.^32^, ^33^, ^34^ In this study, we examined how Si fertilization influences the attractiveness of volatiles from FAW‐infested maize plant to *D. luteipes*, as well as the composition of nocturnal volatile emissions with the aim of identifying compounds that attract this natural enemy. We hypothesized that Si accumulation primes indirect defenses in maize plants, by increasing the production of HIPVs, making them more attractive to the predator.

## 2 MATERIALS AND METHODS

### 2.1 Plant cultivation and Si fertilization

We individually sowed seeds of the conventional maize hybrid 30F53 (Pioneer Seeds, Santa Cruz do Sul, Brazil) into 2‐L polyethylene pots filled with a 2:1 mixture of dark red latosol soil and cattle manure, along with 5 g of nitrogen/phosphorus/potassium (N:P:K, 8:24:12) fertilizer. The Si, in the form of silicic acid (H_4_SiO_2_) (Vertec Química Fina, Duque de Caxias, Brazil), was applied to the soil via drenching. The application rate was adjusted to deliver 0.93 ton Si/ha (equivalent to 2 ton SiO_2_/ha), achieved by dissolving 1.5 g of H_4_SiO_2_ in 150 mL of water.^35^ The Si treatment (+Si plants) was applied to seedlings at the V2 phenological stage 5 days after emergence. Control plants (−Si plants) received the same volume of water without Si. All plants were grown in a glasshouse under natural light in Lavras, Brazil, from November 2020 to July 2021, with no temperature or humidity control (daylight range of 10–13 h, average maximum temperature of 27 ± 1.7 °C, and minimum of 15.7 ± 2.9 °C), and were irrigated on alternate days as needed. In all assays, plants were used at the V4 phenological stage (15–20 days after maize plant emergence). One day before infestation with FAW larvae, plants from all treatments were transferred to the laboratory for acclimation [25 ± 3 °C, 60 ± 10% relative humidity (RH)].

### 2.2 Analysis of Si content

We harvested the shoots of V4 maize plants by cutting them at the base of the stems, then placed them in paper bags and dried them in an oven at 60 °C for 48 h. The dried plant material was ground using a Wiley knife mill (Tecnal Laboratory Equipments, Piracicaba, Brazil) to prepare samples. The Si concentrations in the shoots of four +Si and four −Si plants were determined according to the methodology described by Korndörfer *et al*.^36^

### 2.3 Insects

The earwigs used in the experiments were from a colony maintained in acrylic cages lined with brown paper, kept in a climate‐controlled room (27 ± 2 °C, 70 ± 10% RH, and a 14‐h photophase). To prevent inbreeding depression, field‐collected *D. luteipes* individuals from maize crops in the surroundings areas of Lavras, Minas Gerais, Brazil, were introduced into the laboratory colony on an annual basis. Each cage contained 50‐mL cups with water‐moistened cotton, along with sealed straws lined with cotton at one end to provide shelter for oviposition by earwigs.^37^, ^38^ Fan‐folded papers were also provided as additional shelter, and an artificial diet based on cat food was supplied as food for the earwigs.^39^

The FAW larvae used in the experiments came from a laboratory colony kept in the laboratory in similar climate‐controlled conditions (28 ± 2 °C, 70 ± 10% RH, and a 12‐h photophase). The larvae were fed an artificial diet made from beans, wheat germ, and casein.^39^ Adult moths were housed in cylindrical polyvinyl chloride (PVC) mating cages (40 cm in height × 30 cm in diameter), with white paper lining the inner walls for oviposition. They were fed a sugary solution (10% sugar and 5% ascorbic acid in water). Egg masses were collected and hatched in plastic bags, with the neonate larvae then transferred to the artificial diet.^39^

### 2.4 Plant treatments

The experiments included the following treatments: (i) non‐infested, non‐fertilized plants (−Si plants), (ii) non‐infested, fertilized plants (+Si plants), (iii) non‐fertilized plants infested with FAW larvae (−Si herbivore‐infested plant), and (iv) fertilized plants infested with FAW larvae (+Si herbivore‐infested plants). For the herbivore‐infested treatments, we released 50 first‐instar FAW larvae into the maize plant vessels 24 h before the experiments. The herbivore‐infested plants were then covered with fine‐mesh fabric cages to prevent insect escape. Non‐infested plants were similarly covered to maintain consistent conditions across treatments. Plants from the four treatments were kept under the same laboratory conditions (25 ± 3 °C, 60 ± 10% RH).

### 2.5 Olfactory preference of *D. luteipes* to plant volatile emissions

We evaluated the olfactory preference of *D*. *luteipes* females for nocturnal volatiles emitted by maize plants using a Y‐tube glass olfactometer (18 cm long, 3 cm internal Ø, and an angle of 120° between the arms), following a methodology adapted from Naranjo‐Guevara *et al*.^40^ Maize shoots were enclosed in polyethylene bags (41 cm × 33 cm) with two openings on the distal sides to connect hoses. Charcoal‐filtered and humidified air was pushed into the system and directed to the plants in the bags, then conveyed to the distal arms of the olfactometer. The airflow in the system was set to 0.6 L/min, based on preliminary tests.

We introduced a single unmated adult *D. luteipes* female, 5–15‐day‐old and starved for 48 h before the assay, into the central arm of the olfactometer and observed its behavior for up to 5 min. A choice was recorded if the insect crossed the dashed line located 7.5 cm from the intersection in one of the distal arms. If the insect did not choose an arm within 5 min, it was considered as non‐responsive. Each insect was tested only once, and the glassware was replaced with clean glass after each trial. To avoid positional bias, the sides of lateral arms were reversed after testing each insect. After testing eight insects per pair of plants, the plant pairs were replaced. The assays were conducted in the laboratory (25 ± 2 °C, 60 ± 10% RH) between 7:00 p.m. and 9:30 p.m., during which time *D*. *luteipes* is active for foraging.^40^ Laboratory lights were kept off, and red light was used to visualize the insects. Initially, we evaluated the attractiveness of constitutive emission and HIPVs from maize plants (unfertilized) to earwigs by testing: (i) −Si plants *versus* clean air; (ii) −Si herbivore‐infested plants *versus* clean air; (iii) −Si herbivore‐infested plants *versus* −Si plants. Then, we tested the following treatment combinations: (iv) +Si plants *versus* −Si plants, and (v) +Si herbivore‐infested plants *versus* −Si herbivore‐infested plants. Each olfactometry assay consisted of at least 48 responses using six plant pairs from each treatment.

### 2.6 Chemical characterization of volatile plant profiles

We collected the nocturnal volatile emissions from eight maize plants per treatment (four treatments in total) between 7:00 p.m. and 9:30 p.m., the same time interval of the olfactometer assays, using a push‐pull system. Each maize shoot was individually bagged in polyethylene bags (41 cm × 33 cm) and connected to an air inlet flow of 1.1 L/min per bag. A vacuum pump then pulled air at a rate of 0.8 L/min per bag, which was connected to volatile collection filters. These filters consisted of glass tubes filled with 30 mg of HayeSep‐Q® adsorbent polymer (Hayes Separation Inc., Bandera, TX, USA). After the collection, the filters were eluted with 150 μL of dichloromethane, and the resulting extracts were stored in glass vials at −80 °C until analysis. We added 10 μL of a solution containing 200 ng of *n*‐octane and 400 ng of nonyl acetate as internal standards to each sample. A 2‐μL aliquot of each sample was then injected into a non‐polar capillary column (Rtx‐1, 25 m × 0.25 mm diameter × 0.25 μm wall thickness; Restek, Bellefonte, PA, USA) of a gas chromatography‐flame ionization detector (GC‐FID, Shimadzu GC‐2010; Shimadzu Corp., Kyoto, Japan). Helium served as the carrier gas at a flow rate of 25 cm/s. The column temperature was initially held at 40 °C for 5 min, then increased at a rate of 5 °C/min until it reached 150 °C, before finally increasing to 250 °C at a rate of 20 °C/min, where it was held for 30 min. Representative samples were also analyzed using gas chromatography–mass spectrometry (GC–MS, QP2010 Ultra; Shimadzu Corp.) following a similar method to that used for the GC‐FID analyses. Mass spectra were obtained in a full scan mode (electron impact, 75 eV; ion source temperature: 250 °C) over the range of *m/z* 35–270. Chemical identification of the compounds was performed using retention indices and mass spectra comparison with the NIST11 library and synthetic standards, when available.

### 2.7 Olfactory preference of *D*. *luteipes* for synthetic compounds

We evaluated the olfactory response of *D. luteipes* females to the synthetic neryl acetate (90%; Sigma‐Aldrich, St Louis, MO, USA), a volatile terpene compound exclusively released by +Si herbivore‐infested plants. The compound was diluted in paraffin oil (Sigma‐Aldrich) at three concentrations: the first corresponding to the ratio emitted by +Si herbivore‐infested plants (0.21 ng/μL), the second ten times lower (0.021 ng/μL), and the third ten times higher (2.1 ng/μL). The method used to assess the earwig's olfactory response was similar to that used to test its preference for plant emissions, but it differed in the olfactometer system and the use of dispensers impregnated with neryl acetate solutions. The Y‐tube glass olfactometer was slightly narrower with longer arms (0.9 cm internal diameter, a main arm of 25 cm length, lateral arms of 20 cm length, and a 120° angle between the lateral arms). Each lateral arm of the olfactometer was connected by hoses to 0.3 L glass chambers, with one containing the dispenser (a 3 cm^2^ paper filter) impregnated with 10 μL of the neryl acetate solution, while the other contained a control (i.e., the same volume of paraffin oil in the paper filter). Charcoal‐filtered and humidified air was delivered at a rate of 0.8 L/min per arm. The assays were conducted in the laboratory at the same time of the night and under the same climate conditions, using earwig females of the same age and starvation period as previously described. For each replicate, the filter papers impregnated with the synthetic solution and the control were replaced with new ones. At least 43 earwigs were tested for each concentration of the neryl acetate solution.

### 2.8 Statistical analysis

Initially, Si content data and individual volatile amounts were subjected to the Shapiro–Wilk and Bartlett tests to verify the assumptions of normality and homoscedasticity, respectively. The mean Si contents in the leaves of +Si and −Si plants were compared using a Student's *t*‐test. The choice data of *D*. *luteipes* olfactory preference in response to plant volatile emissions were analyzed using a generalized linear mixed model (GLMM) with a binomial distribution, considering the treatments as fixed variables and plant pairs as random variables. To assess whether *D*. *luteipes* females were attracted to the synthetic compound neryl acetate, we analyzed the data using a generalized linear model (GLM) with a binomial distribution. Model fit quality was evaluated through a semi‐normal plot with a simulation envelope. The amounts of individual volatiles and total content of each chemical group emitted from maize plants were compared using the non‐parametric Wilcoxon test. The composition of plant volatile emissions was analyzed by the principal component analysis (PCA). All analyses were performed in R software^41^ using the following packages: lme4,^42^ emmeans,^43^ and hnp.^44^

## 3 RESULTS

### 3.1 Analysis of Si content

The Si‐fertilized maize plants (+Si) had, on average, approximately twice the Si content [mean ± standard error (SE): 1.132 ± 0.080 g/kg dry leaf tissue) in their leaves compared to unfertilized plants (−Si) (mean ± SE: 0.632 ± 0.015 g/kg dry leaf tissue) (*t* = −5.50, df = 6, *P* = 0.010).

### 3.2 Olfactory preference of *D. luteipes*

**Figure 1:**
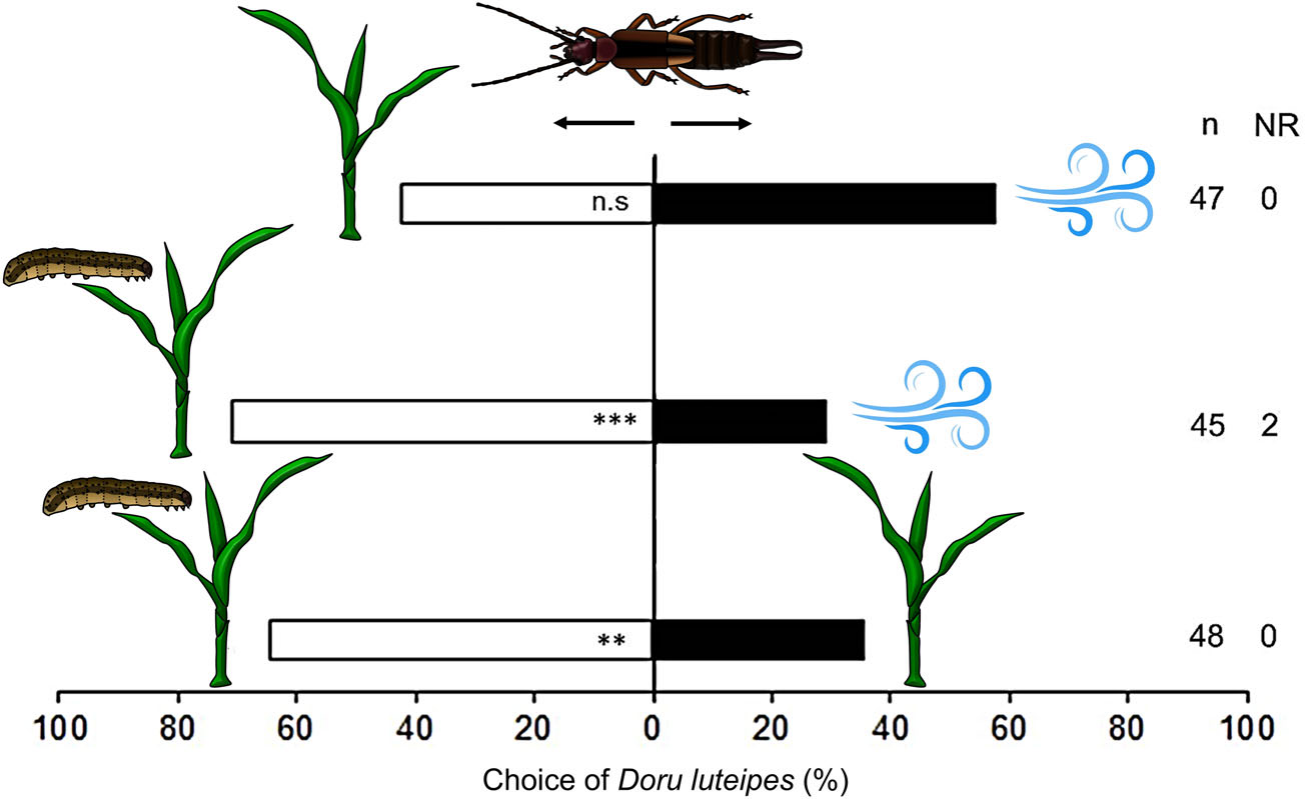
Olfactory response of the predatory earwig *Doru luteipes* to volatiles emitted from non‐fertilized maize plant volatiles. The combinations of odor sources were tested in an olfactometry system and consisted of (i) maize plant *versus* clean air (represented by the wind current icon); (ii) maize plant infested by *Spodoptera frugiperda versus* clean air; and (iii) maize plant infested by *S. frugiperda versus* maize plant. ‘NR’ = the number of ‘non‐responsive’ insects; ‘*n*’ = the number of insects tested; *** = significant difference at 0.1%; ** = significant difference at 1%; ‘n.s’ = not significant (GLMM‐binomial, Wald *χ* ^2^ test).

**Figure 2:**
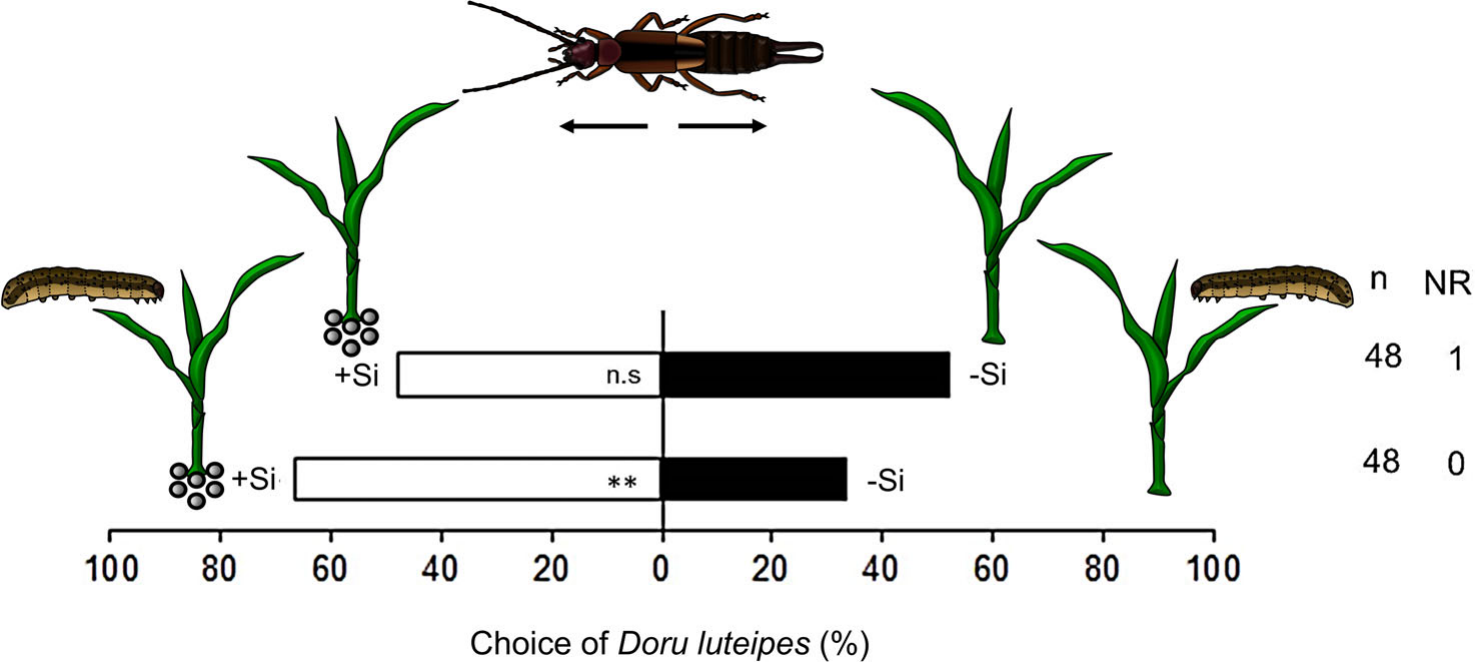
Effect of silicon (Si) fertilization on the olfactory preference of *Doru luteipes* for maize plants volatiles. Tests were performed in an olfactometry system with the odor sources in the following combinations: (i) unfertilized plants (−Si) *versus* Si‐fertilized plants (+Si) plants, and (ii) −Si plants infested by *Spodoptera frugiperda versus* +Si plants infested by *S. frugiperda*. ‘NR’ = number of ‘non‐responsive’ insects, and ‘*n*’ = the number of insects tested. ‘n.s’ = not significant (GLMM‐binomial, Wald *χ* ^2^ test).

Initially, we assessed whether the natural enemy *D. luteipes* was innately attracted to the constitutive and herbivore‐induced plant volatiles from −Si maize plants compared to clean air. While earwigs were not attracted to constitutive maize volatiles, they were attracted to HIPVs (Fig. 1, −Si *versus* clean air: *χ* ^2^ = 0.12, df = 1, *P* = 0.784; −Si herbivore‐infested *versus* clean air: *χ* ^2^ = 12.44, df = 1, *P* < 0.001). In addition, when exposed to volatiles from −Si and −Si herbivore‐infested plants, earwigs clearly preferred HIPVs over constitutive volatile emissions (Fig. 1, −Si *versus* −Si herbivore‐infested: *χ* ^2^ = 9.86, df = 1, *P* = 0.007). Next, we evaluated whether Si fertilization altered the olfactory preference of *D. luteipes* for maize volatiles. Earwigs were not attracted to volatiles released by non‐infested maize plants regardless of Si fertilization (Fig. 2, −Si *versus* +Si, *χ* ^2^ = 0.16, df = 1, *P* = 0.683). However, when exposed simultaneously to volatiles from +Si herbivore‐infested and −Si herbivore‐infested plants, earwigs showed a preference for the HIPVs emitted from +Si plants (Fig. 2, +Si herbivore‐infested *versus* −Si herbivore‐infested, *χ* ^2^ = 10.25, df = 1, *P* = 0.002).

### 3.3 Chemical characterization of the volatile profile of plants

**Figure 3:**
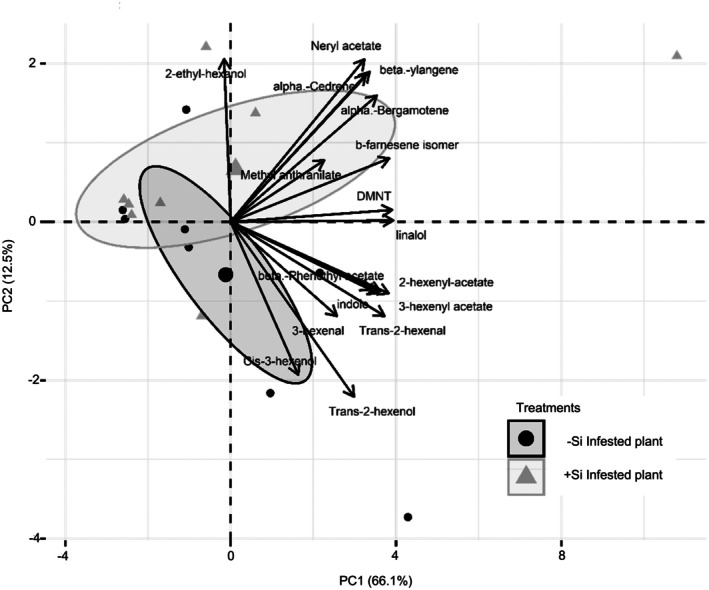
Effect of silicon (Si) fertilization on the composition of herbivore‐induced maize volatiles. Principal component analysis (PCA) of the volatile composition emitted by Si‐fertilized plants infested by *Spodoptera frugiperda* (+Si infested) and non‐fertilized plants infested by *S. frugiperda* (−Si infested plant).

A total of 21 compounds were identified in herbivore‐induced nocturnal volatile emissions from both +Si and −Si plants (Table 1). Multivariate analysis of the volatile composition revealed no clear separation between treatments along the first two principal components (Fig. 3). In contrast, no volatile compounds were detected in the constitutive emissions from non‐infested plants, regardless of Si fertilization. When analyzing the amounts of compounds individually, we found a significant difference in only one compound: the terpene neryl acetate, which was exclusively detected in the emissions from +Si herbivore‐infested plants. However, no significant differences were observed in the total content of each chemical group between treatments.

**Table 1:** Effect of silicon (Si) fertilization on the volatile composition of non‐infested and herbivore‐infested maize plants

Compounds	+Si	−Si	+Si infested	−Si infested	*P*‐Value^†^
*Derivatives of fatty acids*					
3‐Hexenal	—	—	92.21 ± 34.02	109.42 ± 30.78	0.3282
*trans*‐2‐Hexenal	—	—	191.10 ± 90.22	222.62 ± 71.12	0.5737
*cis*‐3‐Hexenol	—	—	71.06 ± 26.60	108.04 ± 52.31	0.5054
*trans*‐2‐Hexenol	—	—	16.39 ± 7.32	27.59 ± 13.29	0.4295
3‐Hexenyl acetate	—	—	253.04 ± 135.82	335.05 ± 95.59	0.3282
2‐Hexenyl acetate	—	—	121.15 ± 79.74	162.60 ± 77.44	0.6454
2‐Methyl pentanol	—	—	6.40 ± 5.30	2 ± 1.05	0.7723
2‐Methyl butanol acetate	—	—	3.4 ± 2.28	2.43 ± 1.31	0.9553
2‐Pentenol acetate	—	—	13.02 ± 6.7	15.44 ± 5.13	0.4418
2‐Ethyl hexanol	—	—	7.9 ± 2.67	8.37 ± 3.47	0.5737
Total			777.66 ± 363.53	993.57 ± 303.52	0.5054
*Aromatic compounds*					
Indole	—	—	41.85 ± 32.31	69.59 ± 30.87	0.2345
Total			41.85 ± 32.31	69.59 ± 30.87	0.2345
*Terpenes*					
Linalool	—	—	113.35 ± 68.55	96.91 ± 39.72	0.7209
Beta‐farnesene isomer	—	—	265.29 ± 139.64	216.16 ± 48.68	0.5737
Alpha‐bergamotene	—	—	100.14 ± 61.77	62.90 ± 15.76	0.7209
DMNT^‡^	—	—	45.60 ± 26.06	39.96 ± 13.45	0.6454
TMTT^§^	—	—	20.36 ± 11.67	22.42 ± 12.25	1
Alpha‐cedrene	—	—	9.03 ± 4.14	3.13 ± 1.82	0.1501
Neryl acetate^*^	—	—	10.53 ± 6.78	0.00 ± 0.00	0.0325
Beta‐ylangene	—	—	32.21 ± 21.89	5.96 ± 1.95	0.6454
Total			596.51 ± 339.04	447.43 ± 114.94	0.7209
*Esters*					
Methyl anthranilate	—	—	8.52 ± 2.75	5.96 ± 1.95	0.4589
Beta‐phenethyl acetate	—	—	18.28 ± 16.02	36.49 ± 16.45	0.5898
Total			26.8 ± 17.11	42.29 ± 18.34	0.7923

*Note*: Amounts of volatile compounds (mean ± standard error ng/g of dry shoot weight) present in the blend emitted by maize plants under different conditions: plants fertilized with silicon (+Si), plants not fertilized with silicon (−Si), plants fertilized with silicon and infested with the fall armyworm *Spodoptera frugiperda* (+Si infested), and not fertilized with silicon and infested with *S. frugiperda* (−Si infested).

^†^ According to non‐parametric Wilcoxon test.

^‡^ DMNT: (E)‐4,8‐dimethyl‐1,3,7‐nonatriene

^§^ TMTT: (E,E)‐4,8,12‐trimethyl‐1,3,7,11‐tridecatetraene.

^*^ significant difference at 5%.

### 3.4 Olfactory preference of *D*. *luteipes* for neryl acetate

**Figure 4:**
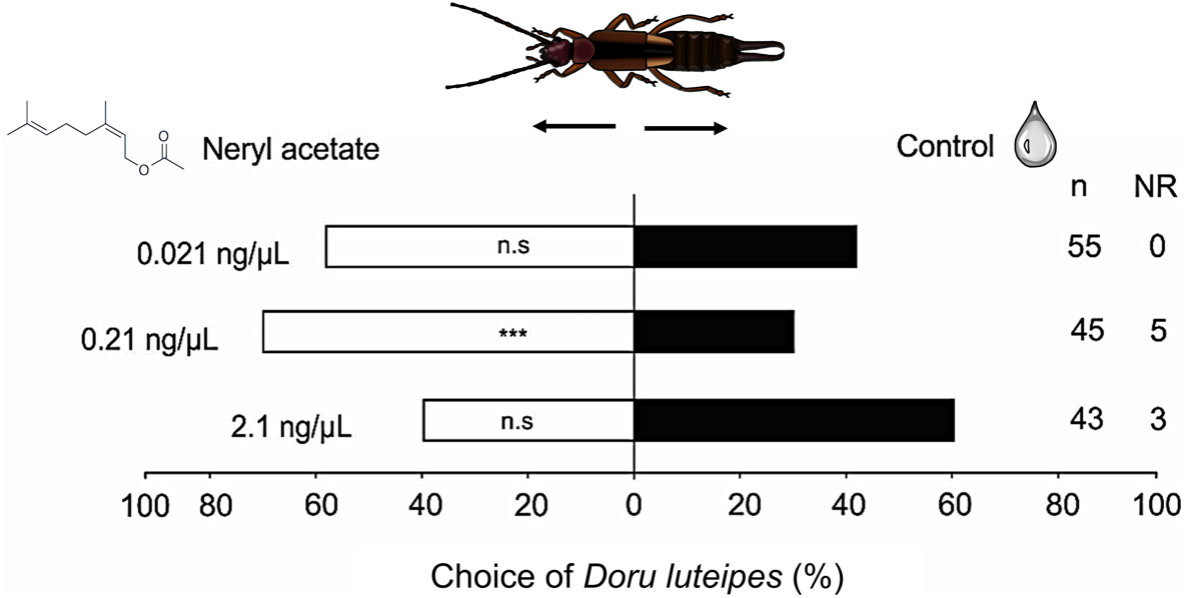
Attractiveness of neryl acetate to the predatory earwig *Doru luteipes*. Olfactory preference was evaluated in an olfactometry system with the combination of odor sources: (i) neryl acetate at 0.021 ng/μL *versus* control (paraffin oil represented by the drop icon), (ii) neryl acetate at 0.21 ng/μL *versus* control, and (iii) neryl acetate at 2.1 ng/μL *versus* control. ‘NR’ represents the number of ‘non‐responsive’ insects, and ‘*n*’ represents the number of replicates. ‘n.s’ = not significant; *** = significant difference at 0.1% (GLM‐binomial, Wald *χ* ^2^ test).

Females of *D. luteipes* were significantly attracted to the synthetic version of neryl acetate at the concentration released by +Si herbivore‐infested maize plants, compared to the control (paraffin oil) (Fig. 4, neryl acetate 0.21 ng/μL *versus* control: *χ* ^2^ = 13.16, df = 1, *P* < 0.001). However, earwigs were not attracted to neryl acetate at concentrations either ten times higher (2.1 ng/μL: *χ* ^2^ = 2.95, df = 1, *P* = 0.085) or ten times lower (0.021 ng/μL: *χ* ^2^ = 3.22, df = 1, *P* = 0.072) (Fig. 4).

## 4 DISCUSSION

The effect of Si fertilization on the priming of herbivore‐induced plant defenses, such as the emission of HIPVs that attract natural enemies of pests, remains poorly understood and underexplored. To date, studies have reported variable effects, with Si accumulation in plants increasing attractiveness to natural enemies through two main ways: (i) by enhancing attractiveness of HIPVs,^26^, ^27^ as a result of changes in the proportions of volatile compounds in the blend^20^, ^27^; or (ii) by inducing the emission of constitutive plant volatiles that attract natural enemies but in the absence of hosts/prey.^21^ Our findings support the former mechanism, indicating that Si accumulation accentuates herbivore‐induced plant response by increasing the attractiveness of HIPVs to predators, while constitutive volatile emissions remain unchanged. However, unlike previous studies,^20^, ^27^ our results suggest that this effect was mediated by a single volatile compound exclusively emitted by Si‐fertilized herbivore‐infested plants: the terpene neryl acetate.

In a previous study, we found that Si accumulation in maize plants negatively impacted oviposition and increased larval mortality of FAW, while also enhancing the attractiveness of diurnal emission of HIPVs to the pirate flower bug *O. insidiosus*.^26^ Here our study focused on the olfactory preference of an important night‐active neotropical predator of FAW to nocturnal plant volatile blends, which have a distinct composition from those emitted during the day, especially concerning terpenes whose synthesis is dependent on photosynthesis.^40^, ^45^, ^46^ In olfactometry tests, we observed that the accumulation of Si in maize plants did not alter the earwig preference to constitutive nocturnal plant volatiles, but increased the attractiveness of volatiles induced by FAW herbivory to the predatory earwig *D*. *luteipes*.

The chemical analysis of plant profiles revealed a drastic difference between the nocturnal emissions of herbivore‐infested and non‐infested maize plants, regardless of Si fertilization. While prior studies have observed that non‐infested maize emits a few volatiles at night,^40^, ^47^ we did not detect any compounds in the nocturnal volatile blend emitted by non‐infested maize plants, which is consistent with the lack of preference shown by *D. luteipes* between +Si and −Si non‐infested plants. Considering the similarity of methods for volatile collection and identification, this difference in the volatile blend among studies stems from the use of different hybrids, as maize exhibits high genetic variability in volatile emissions.^48^, ^49^ Our findings indicated that Si fertilization alone did not induce any change in the constitutive maize volatile emission, unlike those observed in other grass. Oliveira *et al*.^21^ found that Si accumulation in wheat induced the constitutive release of the terpene geranyl acetone, attracting aphid parasitoids, even in the absence of hosts. From a biological control perspective, the attraction of natural enemies to constitutive volatiles may prove detrimental as they will not be rewarded with encounters with prey/hosts, potentially leading to a negative association with these volatiles. Therefore, we expect that Si fertilization in maize is a more promising tactic than in wheat for enhancing biological control, as it does not trigger the emission of constitutive volatiles that attract predators, as demonstrated in our study for *D. luteipes* and previously for the minute pirate bug *O. insidiosus*.^26^

Fatty acid derivatives (FADs) and terpenes were the dominant chemical groups in the volatile blend emitted by plants infested with FAW, regardless of the Si regime. Previous studies suggest that nocturnal emissions of FADs play a role in attracting the predator *D*. *luteipes*.^40^, ^50^ Since herbivore‐infested plants released a complex blend of compounds and both herbivore‐infested treatments emitted similar amounts of FADs, our results do not allow us to infer the relevance of this group in recruiting *D. luteipes* to herbivore‐infested plants. However, we found that the terpene neryl acetate, the only compound that differed between the volatile blends of +Si and −Si herbivore‐infested plants, was responsible for the enhanced attractiveness to the predatory earwig. We confirmed the role of neryl acetate in earwig attraction through olfactometry tests using this compound alone as the odor source. Interestingly, the attraction of *D*. *luteipes* to neryl acetate was dose‐dependent, with the predator being attracted only to the concentration equivalent to that emitted by Si‐fertilized infested plants.

Neryl acetate is commonly found in volatile blends, either as a major or minor component, emitted by various plants across different families, such as Rutaceae,^51^ Pinaceae,^52^ Lamiaceae,^53^ Rosaceae,^54^ and Asteraceae.^55^ Previously, Tamiru *et al*.^56^ identified neryl acetate as an oviposition‐induced plant volatile in maize varieties, but without a significant role in attracting natural enemies. Here, we report for the first time the attractiveness of neryl acetate to a natural enemy, the predatory earwig *D*. *luteipes*. The attractiveness of volatile blends from herbivore‐infested treatments to the predatory earwig, regardless of Si fertilization, is likely influenced by the presence and ratios of other compounds in the blend, as −Si herbivore‐infested were preferred over −Si intact plants. The literature suggests that natural enemies are attracted by the combination of several volatile compounds rather than a single compound.^57^, ^58^ Although it is unlikely that neryl acetate alone would be as attractive as the full blend from +Si herbivore‐infested plant, the fact that the only difference between the HIPV blends from +Si and −Si plants was the presence of neryl acetate – and its demonstrated attractiveness when tested in isolation – supports its role as an important attractant to the earwig.

Since early consumption of FAW neonate larvae on Si‐fertilized maize plants is similar to that in non‐fertilized maize plants,^26^ the detection of the terpene neryl acetate in Si‐fertilized infested plants suggests that the induced response was intensified. Ye *et al*.^15^ showed that Si fertilization increases levels of JA after herbivory, enhancing plant resistance and acting as a priming agent for plant defenses, including the emission of HIPVs.^15^, ^20^, ^59^, ^60^ Thus, the greater attractiveness of volatiles emitted by Si‐fertilized plants may be due to a priming effect of Si on the JA signaling pathway, leading to faster and stronger responses,^15^ such as changes in the chemical composition of plant volatiles to make them attractive to natural enemies. The priming effect of Si accumulation in maize defenses against FAW is also supported by levels of biochemical markers of plant defenses, such as reactive oxygen species and antioxidant enzymes.^61^

Our study demonstrated that Si fertilization altered the nocturnal volatile profile of maize plants induced by herbivory of the key pest FAW, leading to the emission of the terpene neryl acetate, which rendered plants more attractive to the predatory earwig *D*. *luteipes*. Although *D*. *luteipes* is considered a generalist and omnivorous predator, it is one of the main natural enemies of immature FAW in maize fields in the neotropical region.^32^, ^62^ Along with our previous findings,^26^ our results indicate that Si fertilization in maize fields is a promising strategy for FAW control, as it may directly impact the pest by reducing egg laying and neonate larvae survival, and indirectly by recruiting both day‐active and night‐active predators to FAW‐infested plants. Moreover, our results provide insights into the potential application of synthetic neryl acetate as an attractant for *D. luteipes*. However, its biological activity on the behavior of FAW ovipositing moths remains unknown, and we encourage studies to determine whether neryl acetate acts as a repellent or attract for the moths. Furthermore, Si fertilization is known to confer increased plant resistance against FAW in the field,^31^, ^32^, ^33^ but our study suggests that this tactic may have an even greater impact by enhancing the ability of natural enemies to locate prey‐infested plants. Further field studies are required to evaluate the compatibility between Si fertilization and biological control tactics, specifically investigating whether Si fertilization effectively enhances FAW regulation through the combined benefits of increased plant resistance and biological control by entomophagous insects.

## CONFLICT OF INTEREST STATEMENT

The authors declare no conflict of interest.

## Data Availability

The data that support the findings of this study are available from the corresponding author upon reasonable request.
